# Supplementary material for: Two New Cases of Hypertrophic Cardiomyopathy and Skeletal Muscle Features Associated with ALPK3 Homozygous and Compound Heterozygous Variants
Source: Genes (Basel). 2020 Oct 15;11(10):1201. doi: 10.3390/genes11101201 (PMC7602582; doi:10.3390/genes11101201)
Supplement: Supplementary file 1 [file genes-11-01201-s001.zip › genes-964740- supplementary/ALPK3_genes-proofread/Supplementary Tables_genes.docx]

Supplementary Table 1.

List of studied genes for Patient 1 (Almazov_CardioMyoPathy_Arrhythmia_Noonan Design ID: 27291-1393420132)

108 Target IDs resolved to 108 targets comprising 2159 regions.

| **TargetID** | **Interval** | **Coverage** |
| --- | --- | --- |
| ABCC9 | chr12:21953968-22089618 | 100.00 |
| ACTC1 | chr15:35082603-35087019 | 100.00 |
| ACTN2 | chr1:236849964-236925929 | 99.82 |
| AKAP9 | chr7:91570404-91739483 | 99.93 |
| ANK2 | chr4:113825640-114309894 | 100.00 |
| ANKRD1 | chr10:92672613-92680794 | 100.00 |
| BAG3 | chr10:121411178-121436804 | 100.00 |
| BRAF | chr7:140426284-140624513 | 100.00 |
| CACNA1C | chr12:2162719-2800375 | 99.95 |
| CACNA2D1 | chr7:81579698-82072785 | 100.00 |
| CACNB2 | chr10:18429656-18828663 | 99.78 |
| CALM1 | chr14:90863565-90871071 | 100.00 |
| CALR3 | chr19:16589932-16606950 | 100.00 |
| CASQ2 | chr1:116243852-116311172 | 100.00 |
| CAV3 | chr3:8775553-8787563 | 100.00 |
| CBL | chr11:119077118-119170501 | 100.00 |
| CRYAB | chr11:111779478-111782458 | 100.00 |
| CSRP3 | chr11:19204207-19214005 | 99.85 |
| DES | chr2:220283175-220290722 | 100.00 |
| DMD | chrX:31132798-33357392 | 99.92 |
| DMPK | chr19:46273729-46285639 | 100.00 |
| DSC2 | chr18:28647971-28681944 | 100.00 |
| DSG2 | chr18:29078205-29126716 | 100.00 |
| DSP | chr6:7542139-7586121 | 100.00 |
| DTNA | chr18:32335931-32470414 | 100.00 |
| EMD | chrX:153607835-153609567 | 100.00 |
| EYA4 | chr6:133595909-133852374 | 100.00 |
| FHL1 | chrX:135251952-135292194 | 100.00 |
| FHL2 | chr2:105977730-106015563 | 100.00 |
| FKTN | chr9:108337304-108402414 | 99.68 |
| FXN | chr9:71650689-71714860 | 100.00 |
| GAA | chr17:78078376-78093140 | 100.00 |
| GLA | chrX:100652787-100662901 | 100.00 |
| GPD1L | chr3:32148194-32207412 | 100.00 |
| HCN4 | chr15:73614812-73660621 | 100.00 |
| HRAS | chr11:532626-534332 | 100.00 |
| ILK | chr11:6625492-6631852 | 100.00 |
| JPH2 | chr20:42743426-42815355 | 100.00 |
| JUP | chr17:39775836-39928116 | 100.00 |
| KCND3 | chr1:112318689-112525358 | 100.00 |
| KCNE1 | chr21:35821533-35821942 | 100.00 |
| KCNE1L | chrX:108867811-108868259 | 100.00 |
| KCNE2 | chr21:35742768-35743159 | 100.00 |
| KCNE3 | chr11:74168287-74168618 | 100.00 |
| KCNH2 | chr7:150642443-150675011 | 100.00 |
| KCNJ2 | chr17:68171171-68172474 | 100.00 |
| KCNJ5 | chr11:128781159-128786636 | 100.00 |
| KCNJ8 | chr12:21918647-21926560 | 100.00 |
| KCNQ1 | chr11:2466319-2869243 | 100.00 |
| KRAS | chr12:25362719-25398328 | 100.00 |
| LAMA4 | chr6:112430630-112575362 | 100.00 |
| LAMP2 | chrX:119562329-119603034 | 100.00 |
| LDB3 | chr10:88428439-88492743 | 100.00 |
| LMNA | chr1:156084700-156109640 | 99.71 |
| MAP2K1 | chr15:66679676-66782963 | 100.00 |
| MAP2K2 | chr19:4090586-4123882 | 100.00 |
| MRPL3 | chr3:131181557-131221837 | 100.00 |
| MYBPC3 | chr11:47353412-47374208 | 100.00 |
| MYH6 | chr14:23851239-23876442 | 99.44 |
| MYH7 | chr14:23882053-23902951 | 99.55 |
| MYL2 | chr12:111348871-111358343 | 88.61 |
| MYL3 | chr3:46899724-46904890 | 100.00 |
| MYLK2 | chr20:30407374-30421610 | 100.00 |
| MYOM1 | chr18:3067250-3215231 | 100.00 |
| MYOZ2 | chr4:120057671-120107365 | 100.00 |
| MYPN | chr10:69866472-69970222 | 100.00 |
| NEBL | chr10:21074666-21462772 | 100.00 |
| NEXN | chr1:78381782-78408608 | 100.00 |
| NF1 | chr17:29422318-29705959 | 99.63 |
| NOS1AP | chr1:162039958-162353331 | 100.00 |
| NRAS | chr1:115251146-115258791 | 100.00 |
| PDLIM3 | chr4:186423438-186456598 | 99.45 |
| PKP2 | chr12:32945348-33049675 | 100.00 |
| PLN | chr6:118880075-118880253 | 100.00 |
| PRKAG2 | chr7:151254277-151573715 | 99.87 |
| PSEN1 | chr14:73614718-73686007 | 100.00 |
| PSEN2 | chr1:227068337-227083290 | 100.00 |
| PTPN11 | chr12:112856906-112942578 | 100.00 |
| RAF1 | chr3:12626003-12660230 | 99.57 |
| RANGRF | chr17:8192097-8193264 | 100.00 |
| RBM20 | chr10:112404203-112595746 | 100.00 |
| RYR2 | chr1:237205812-237995957 | 99.70 |
| SCN1B | chr19:35521715-35530615 | 99.64 |
| SCN3B | chr11:123504841-123524519 | 100.00 |
| SCN4B | chr11:118007732-118023398 | 100.00 |
| SCN5A | chr3:38591802-38674808 | 99.73 |
| SCO2 | chr22:50962030-50962850 | 100.00 |
| SDHA | chr5:218461-256545 | 97.59 |
| SGCD | chr5:155756577-156186411 | 100.00 |
| SHOC2 | chr10:112724107-112771586 | 100.00 |
| SLC25A3 | chr12:98987747-98995316 | 100.00 |
| SLMAP | chr3:57743369-57913125 | 100.00 |
| SNTA1 | chr20:31996303-32031436 | 100.00 |
| SOS1 | chr2:39212955-39347573 | 99.65 |
| SPRED1 | chr15:38545377-38643875 | 100.00 |
| TAZ | chrX:153640171-153649353 | 100.00 |
| TCAP | chr17:37821603-37822372 | 100.00 |
| TGFB3 | chr14:76425520-76447246 | 100.00 |
| TMEM43 | chr3:14166684-14183305 | 100.00 |
| TMPO | chr12:98909537-98941646 | 100.00 |
| TNNC1 | chr3:52485281-52488041 | 100.00 |
| TNNI3 | chr19:55663192-55668967 | 100.00 |
| TNNT2 | chr1:201328328-201342392 | 100.00 |
| TPM1 | chr15:63335019-63363381 | 100.00 |
| TRDN | chr6:123539736-123957930 | 98.85 |
| TRPM4 | chr19:49661114-49714765 | 100.00 |
| TTN | chr2:179391729-179682294 | 98.80 |
| VCL | chr10:75757956-75877977 | 100.00 |

Supplementary Table 2

List of studied genes. (Almazov_Comprehensive_Cardiac_Panel)

173 Target IDs resolved to 172 targets comprising 3271 regions.

| **TargetID** | **Interval** | **Coverage** |
| --- | --- | --- |
| ABCC9 | chr12:21801034-21936684 | 100.0 |
| ACADVL | chr17:7217136-7225107 | 100.0 |
| ACTA1 | chr1:229431489-229433125 | 100.0 |
| ACTC1 | chr15:34790402-34794818 | 100.0 |
| ACTN2 | chr1:236686664-236762629 | 100.0 |
| ACVR2B | chr3:38454313-38483342 | 100.0 |
| AGK | chr7:141555457-141652934 | 100.0 |
| AKAP9 | chr7:91941090-92110169 | 100.0 |
| ALPK3 | chr15:84816837-84868466 | 100.0 |
| ANK2 | chr4:112904484-113381650 | 100.0 |
| ANKRD1 | chr10:90912856-90921037 | 100.0 |
| ANO5 | chr11:22193483-22279775 | 100.0 |
| BAG3 | chr10:119651666-119677292 | 100.0 |
| BRAF | chr7:140726484-140924713 | 100.0 |
| CACNA1C | chr12:1971053-2691209 | 100.0 |
| CACNA2D1 | chr7:81950382-82443469 | 100.0 |
| CACNB2 | chr10:18140727-18539734 | 100.0 |
| CALM1 | chr14:90397221-90404727 | 100.0 |
| CALM2 | chr2:47160766-47176551 | 100.0 |
| CALM3 | chr19:46601425-46609163 | 100.0 |
| CALR3 | chr19:16479121-16496139 | 100.0 |
| CASQ2 | chr1:115701231-115768551 | 100.0 |
| CAV3 | chr3:8733867-8745877 | 100.0 |
| CBL | chr11:119206408-119308087 | 100.0 |
| CDH2 | chr18:27952143-28177032 | 100.0 |
| CMYA5 | chr5:79689898-79799626 | 100.0 |
| CRELD1 | chr3:9934429-9944589 | 100.0 |
| CRYAB | chr11:111908754-111911734 | 100.0 |
| CSRP3 | chr11:19182660-19192458 | 100.0 |
| CTNNA3 | chr10:65920320-67648780 | 100.0 |
| DES | chr2:219418453-219426000 | 100.0 |
| DMD | chrX:31121823-33339275 | 100.0 |
| DMPK | chr19:45770471-45782381 | 100.0 |
| DNAAF1 | chr16:84145431-84178416 | 100.0 |
| DNAAF3 | chr19:55159052-55166658 | 100.0 |
| DPP6 | chr7:153887674-154892490 | 100.0 |
| DSC2 | chr18:31068005-31101981 | 100.0 |
| DSG2 | chr18:31498242-31546753 | 100.0 |
| DSP | chr6:7541906-7585888 | 100.0 |
| DTNA | chr18:34755967-34890450 | 100.0 |
| DYSF | chr2:71453989-71686502 | 100.0 |
| EMD | chrX:154379475-154381207 | 100.0 |
| EYA4 | chr6:133274771-133531236 | 100.0 |
| FHL1 | chrX:136196793-136210035 | 100.0 |
| FHL2 | chr2:105361273-105399555 | 100.0 |
| FHOD3 | chr18:36297826-36779540 | 100.0 |
| FKRP | chr19:46755441-46756948 | 100.0 |
| FKTN | chr9:105575023-105640133 | 100.0 |
| FLNA | chrX:154348839-154371255 | 100.0 |
| FLNC | chr7:128830628-128858533 | 100.0 |
| FXN | chr9:69035773-69099944 | 100.0 |
| GAA | chr17:80104577-80119341 | 100.0 |
| GATA4 | chr8:11708303-11758485 | 100.0 |
| GATA5 | chr20:62464826-62475531 | 100.0 |
| GATA6 | chr18:22171135-22200833 | 100.0 |
| GATAD1 | chr7:92447720-92456572 | 100.0 |
| GDF1 | chr19:18868587-18870317 | 100.0 |
| GJA5 | chr1:147758152-147759248 | 100.0 |
| GLA | chrX:101397799-101407913 | 100.0 |
| GPD1L | chr3:32106702-32165920 | 100.0 |
| HAND1 | chr5:154475796-154478018 | 100.0 |
| HCN4 | chr15:73322471-73368280 | 100.0 |
| HFE | chr6:26087431-26094421 | 100.0 |
| HRAS | chr11:532626-534332 | 100.0 |
| ILK | chr11:6604262-6610621 | 100.0 |
| ISPD | chr7:16091685-16421332 | 100.0 |
| JPH2 | chr20:44114786-44186715 | 100.0 |
| JUP | chr17:41755734-41771864 | 100.0 |
| KCNA5 | chr12:5044138-5045999 | 100.0 |
| KCND3 | chr1:111776067-111982736 | 100.0 |
| KCNE1 | chr21:34449235-34449644 | 100.0 |
| KCNE2 | chr21:34370469-34370860 | 100.0 |
| KCNE3 | chr11:74457242-74457573 | 100.0 |
| KCNE5 | chrX:109624582-109625030 | 100.0 |
| KCNH2 | chr7:150945355-150977923 | 100.0 |
| KCNJ2 | chr17:70175030-70176333 | 100.0 |
| KCNJ5 | chr11:128911264-128916741 | 100.0 |
| KCNJ8 | chr12:21765713-21773626 | 100.0 |
| KCNQ1 | chr11:2444676-2848013 | 100.0 |
| KRAS | chr12:25209785-25245394 | 100.0 |
| LAMA4 | chr6:112109427-112254160 | 100.0 |
| LAMP2 | chrX:120428474-120469179 | 100.0 |
| LDB3 | chr10:86668682-86732986 | 100.0 |
| LEFTY2 | chr1:225937411-225941150 | 100.0 |
| LMNA | chr1:156114909-156139849 | 100.0 |
| LMOD3 | chr3:69109085-69122396 | 100.0 |
| LRRC10 | chr12:69609995-69610848 | 100.0 |
| LZTR1 | chr22:20982362-20997358 | 100.0 |
| MAP2K1 | chr15:66387338-66490625 | 100.0 |
| MAP2K2 | chr19:4090588-4123885 | 100.0 |
| MIB1 | chr18:21741574-21864676 | 100.0 |
| MMP21 | chr10:125766652-125775831 | 100.0 |
| MRAS | chr3:138372874-138402279 | 100.0 |
| MYBPC3 | chr11:47331861-47352657 | 100.0 |
| MYBPHL | chr1:109294229-109307001 | 100.0 |
| MYH6 | chr14:23382030-23407233 | 100.0 |
| MYH7 | chr14:23412844-23433742 | 100.0 |
| MYL2 | chr12:110911067-110920539 | 100.0 |
| MYL3 | chr3:46858234-46863400 | 100.0 |
| MYL4 | chr17:47209413-47223052 | 100.0 |
| MYLK2 | chr20:31819571-31833807 | 100.0 |
| MYOF | chr10:93306953-93482204 | 100.0 |
| MYOM1 | chr18:3067252-3215233 | 100.0 |
| MYOT | chr5:137870642-137887395 | 100.0 |
| MYOZ2 | chr4:119136516-119186210 | 100.0 |
| MYPN | chr10:68106715-68210465 | 100.0 |
| NEBL | chr10:20785737-21173843 | 100.0 |
| NEXN | chr1:77916097-77942923 | 100.0 |
| NF1 | chr17:31095198-31378941 | 100.0 |
| NKX2-5 | chr5:173232559-173235093 | 100.0 |
| NKX2-6 | chr8:23702441-23706608 | 100.0 |
| NPPA | chr1:11845993-11847694 | 100.0 |
| NRAS | chr1:114708525-114716170 | 100.0 |
| NUP155 | chr5:37291890-37370987 | 100.0 |
| PDLIM3 | chr4:185502284-185535444 | 100.0 |
| PKD1L1 | chr7:47775133-47948450 | 100.0 |
| PKP2 | chr12:32792414-32896741 | 100.0 |
| PLEC | chr8:143916167-143975379 | 100.0 |
| PLEKHM2 | chr1:15684549-15733944 | 100.0 |
| PLN | chr6:118558912-118559090 | 100.0 |
| PPA2 | chr4:105369715-105474060 | 100.0 |
| PPP1CB | chr2:28752115-28799313 | 100.0 |
| PRDM16 | chr1:3069250-3433821 | 100.0 |
| PRKAG2 | chr7:151557191-151876630 | 100.0 |
| PSEN1 | chr14:73148010-73219299 | 100.0 |
| PSEN2 | chr1:226880636-226895589 | 100.0 |
| PTPN11 | chr12:112419102-112504774 | 100.0 |
| RAF1 | chr3:12584504-12618731 | 100.0 |
| RANGRF | chr17:8288779-8289946 | 100.0 |
| RBM20 | chr10:110644445-110835988 | 100.0 |
| RIT1 | chr1:155900378-155910895 | 100.0 |
| RRAS | chr19:49635566-49640108 | 100.0 |
| RYR2 | chr1:237042512-237832657 | 100.0 |
| SALL4 | chr20:51784255-51802418 | 100.0 |
| SCN10A | chr3:38697339-38794020 | 100.0 |
| SCN1B | chr19:35030811-35039711 | 100.0 |
| SCN2B | chr11:118166877-118176441 | 100.0 |
| SCN3B | chr11:123634133-123653811 | 100.0 |
| SCN4B | chr11:118137017-118152683 | 100.0 |
| SCN5A | chr3:38550311-38633317 | 100.0 |
| SCNN1G | chr16:23186262-23215479 | 100.0 |
| SDHA | chr5:218346-256430 | 100.0 |
| SGCD | chr5:156329567-156759400 | 100.0 |
| SHOC2 | chr10:110964349-111011828 | 100.0 |
| SLMAP | chr3:57757642-57927398 | 100.0 |
| SNTA1 | chr20:33408497-33443630 | 100.0 |
| SOS1 | chr2:38985814-39120432 | 100.0 |
| SOS2 | chr14:50118334-50231293 | 100.0 |
| SPEG | chr2:219434968-219498199 | 100.0 |
| SPRED1 | chr15:38253176-38351674 | 100.0 |
| SYNE1 | chr6:152122426-152628341 | 100.0 |
| SYNM | chr15:99105190-99133068 | 100.0 |
| SYNPO2L | chr10:73646708-73655932 | 100.0 |
| TAZ | chrX:154411834-154421014 | 100.0 |
| TBX20 | chr7:35202420-35253630 | 100.0 |
| TBX5 | chr12:114355522-114403908 | 100.0 |
| TCAP | chr17:39665350-39666119 | 100.0 |
| TECRL | chr4:64277025-64409361 | 100.0 |
| TGFB3 | chr14:75959177-75980903 | 100.0 |
| TMEM43 | chr3:14125184-14141805 | 100.0 |
| TMPO | chr12:98515858-98547868 | 100.0 |
| TNNC1 | chr3:52451265-52454025 | 100.0 |
| TNNI3 | chr19:55151824-55157599 | 100.0 |
| TNNI3K | chr1:74235442-74543992 | 100.0 |
| TNNT2 | chr1:201359200-201373264 | 100.0 |
| TPM1 | chr15:63042820-63071182 | 100.0 |
| TRDN | chr6:123218591-123636785 | 100.0 |
| TRPM4 | chr19:49157857-49211508 | 100.0 |
| TTN | chr2:178527002-178804652 | 100.0 |
| TTR | chr18:31591893-31598685 | 100.0 |
| VCL | chr10:73998198-74118179 | 100.0 |
| ZIC3 | chrX:137566682-137577281 | 100.0 |

Supplementary Table 3

List of 220 genes associated with cardiac phenotypes

Panel version: Cardiology 3.0 (Date: 2020-02-21), Clinical Genetics, Karolinska Institute

| **Gene name** |
| --- |
| AARS2 |
| ABCC6 |
| ABCC9 |
| ACAD9 |
| ACADVL |
| ACTA1 |
| ACTA2 |
| ACTC1 |
| ACTN2 |
| AGK |
| AGL |
| AGPAT2 |
| AKAP9 |
| ALMS1 |
| ALPK3 |
| ANK2 |
| ANKRD1 |
| ANO5 |
| APOA1 |
| ATPAF2 |
| BAG3 |
| BRAF |
| CACNA1C |
| CACNA2D1 |
| CACNB2 |
| CALM1 |
| CALM2 |
| CALM3 |
| CALR3 |
| CAPN3 |
| CASQ2 |
| CAV3 |
| CBL |
| CDH2 |
| CHRM2 |
| COX15 |
| CPT2 |
| CRYAB |
| CSRP3 |
| CTNNA3 |
| DBH |
| DES |
| DMD |
| DNAJC19 |
| DOLK |
| DPP6 |
| DSC2 |
| DSG2 |
| DSP |
| DTNA |
| DYSF |
| EEF1A2 |
| ELAC2 |
| EMD |
| ENPP1 |
| EPG5 |
| ETFA |
| ETFB |
| ETFDH |
| EYA4 |
| FAH |
| FBX032 |
| FHL1 |
| FHOD3 |
| FKRP |
| FKTN |
| FLNC |
| FOXD4 |
| FOXRED1 |
| FXN |
| GAA |
| GATA4 |
| GATA5 |
| GATA6 |
| GATAD1 |
| GBE1 |
| GFM1 |
| GJA1 |
| GJA5 |
| GLA |
| GLB1 |
| GMPPB |
| GPD1L |
| GTPBP3 |
| GUSB |
| HADHA |
| HAND1 |
| HAND2 |
| HCN4 |
| HFE |
| HRAS |
| ISPD |
| JPH2 |
| JUP |
| KCNA5 |
| KCND3 |
| KCNE1 |
| KCNE2 |
| KCNE3 |
| KCNE5 |
| KCNH2 |
| KCNJ2 |
| KCNJ5 |
| KCNJ8 |
| KCNQ1 |
| KRAS |
| LAMA2 |
| LAMP2 |
| LARGE1 |
| LDB3 |
| LMNA |
| LRRC10 |
| LTZR1 |
| MAP2K1 |
| MAP2K2 |
| MLYCD |
| MRPL3 |
| MRPL44 |
| MRPS22 |
| MTO1 |
| MYBPC3 |
| MYBPHL |
| MYH6 |
| MYH7 |
| MYL2 |
| MYL3 |
| MYL4 |
| MYLK2 |
| MYOT |
| MYOZ2 |
| MYPN |
| NDUFAF2 |
| NEBL |
| NEXN |
| NF1 |
| NKX2-5 |
| NOS1AP |
| NPPA |
| NRAS |
| NUP155 |
| PARS2 |
| PCCA |
| PCCB |
| PDLIM3 |
| PKP2 |
| PLEC |
| PLEKHM2 |
| PLN |
| PNPLA2 |
| POMT1 |
| PPA2 |
| PPP1CB |
| PRDM16 |
| PRKAG2 |
| PTPN11 |
| RAF1 |
| RANGRF |
| RASA2 |
| RBCK1 |
| RBM20 |
| RIT1 |
| RMND1 |
| RRAS |
| RYR2 |
| SALL4 |
| SCN10A |
| SCN1B |
| SCN2B |
| SCN3B |
| SCN4B |
| SCN5A |
| SCNN1B |
| SCNN1G |
| SCO2 |
| SDHA |
| SELENON |
| SGCA |
| SGCB |
| SGCD |
| SGCG |
| SHOC2 |
| SLC22A5 |
| SLC25A20 |
| SLC25A3 |
| SLC25A4 |
| SLMAP |
| SMCHD1 |
| SNTA1 |
| SOS1 |
| SOS2 |
| SPEG |
| SPRED1 |
| TAB2 |
| TAZ |
| TBX20 |
| TBX5 |
| TCAP |
| TECRL |
| TGFB3 |
| TMEM43 |
| TMEM70 |
| TMPO |
| TNNC1 |
| TNNI3 |
| TNNI3K |
| TNNT2 |
| TOR1AIP1 |
| TPM1 |
| TRDN |
| TRIM32 |
| TRPM4 |
| TSFM |
| TTN |
| TTR |
| TXNRD2 |
| VARS2 |
| VCL |
| VCP |
| VPS13A |
| XK |

Supplementary Table 4

Рrimers for verification of *ALPK3* variants by Sanger sequencing

| **Primer** | **Sequence (5´-3´)**  **including standard M13 primers** | **PCR fragment size, bp** | **Tm°C** |
| --- | --- | --- | --- |
| ALPK3_05_F | **TGTAAAACGACGGCCAGT**GACATGTACCTGGAGAACACCC | 521 | 61 |
| ALPK3_05_R | **CAGGAAACAGCTATGACC**CCCCACCCCTTTCTATAAATAGCC |  |  |
| ALPK3_06_F | **TGTAAAACGACGGCCAGT**TGATGAGTTCTGCCCCAACACT | 353 | 62 |
| ALPK3_06_R | **CAGGAAACAGCTATGACC**GTGGTTGTTCTCCACACGCTC |  |  |
| ALPK3_10_F | **TGTAAAACGACGGCCAGT**CTTAATGTGCCCAAAGAGCAGGC | 620 | 62,5 |
| ALPK3_10_R | **CAGGAAACAGCTATGACC**CTCTCTGAGGCCAAGCATCTGAA |  |  |
