# Supplementary material for: Two New Cases of Hypertrophic Cardiomyopathy and Skeletal Muscle Features Associated with ALPK3 Homozygous and Compound Heterozygous Variants
Source: Genes (Basel). 2020 Oct 15;11(10):1201. doi: 10.3390/genes11101201 (PMC7602582; doi:10.3390/genes11101201)
Supplement: Supplementary file 1 [file genes-11-01201-s001.zip › genes-964740- supplementary/ALPK3_genes-proofread/Supplementary_figure.docx]

Supplementary Figure 1.

Histological staining of myocardial biopsy of Patient 2

A B


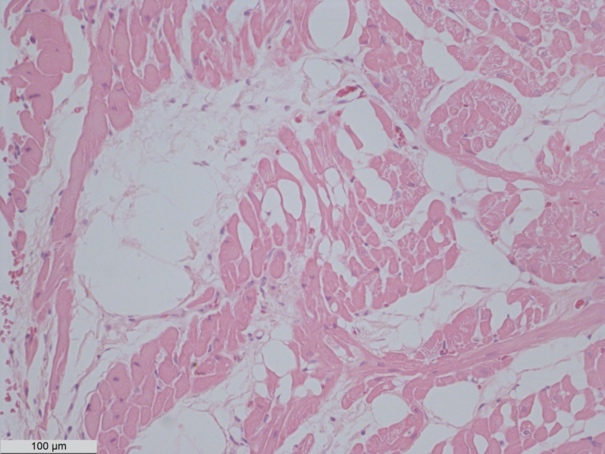

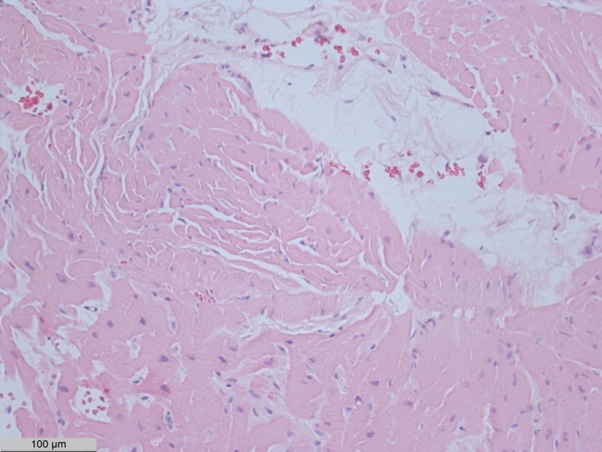


C D


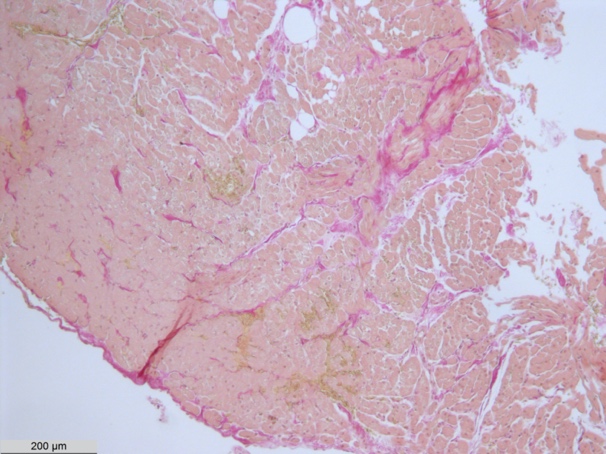

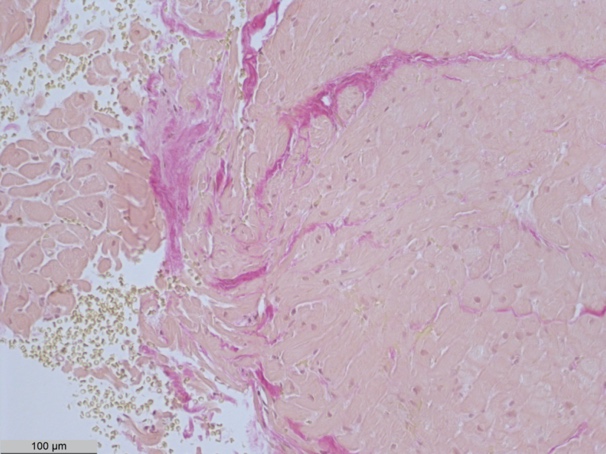


(A,B) Hematoxylin-eosin staining. Fibro-fatty replacement, fiber hypertrophy and nuclear size variability.

(C,D) vanGieson staining. Diffuse areas of fibrosis.
