# Supplementary figures and images for: Two New Cases of Hypertrophic Cardiomyopathy and Skeletal Muscle Features Associated with ALPK3 Homozygous and Compound Heterozygous Variants
Source: Genes (Basel). 2020 Oct 15;11(10):1201. doi: 10.3390/genes11101201 (PMC7602582; doi:10.3390/genes11101201)

Variant Filtering Flow Chart

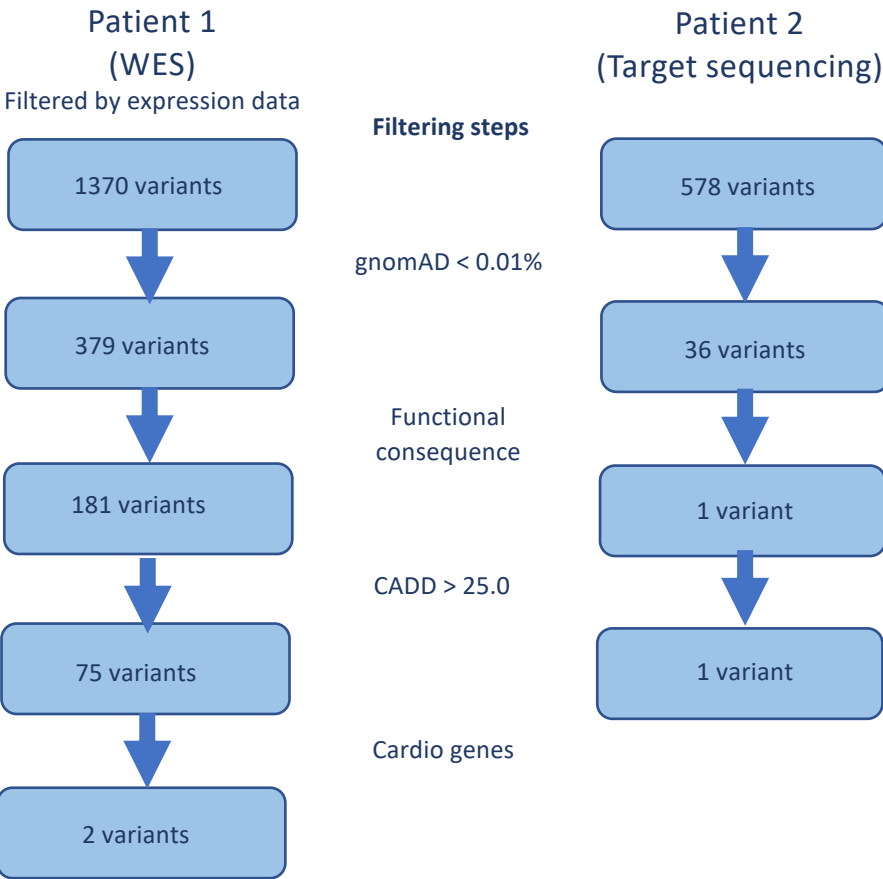

Supplement: Supplementary file 1 [file genes-11-01201-s001.zip › genes-964740- supplementary/ALPK3_genes-proofread/Figure 1.pdf]

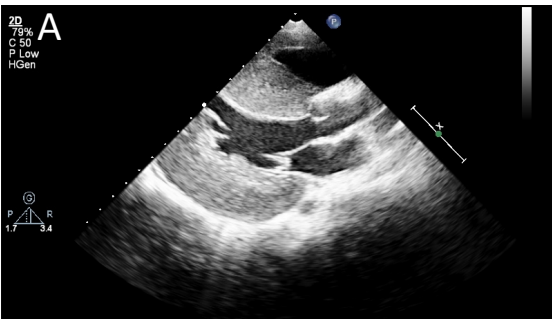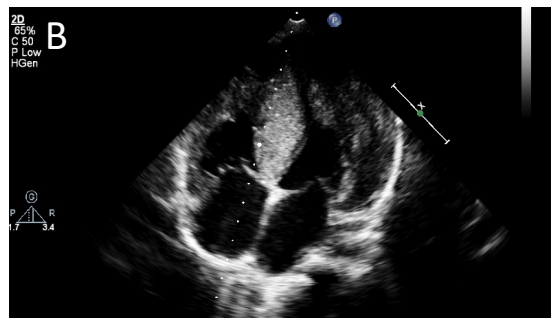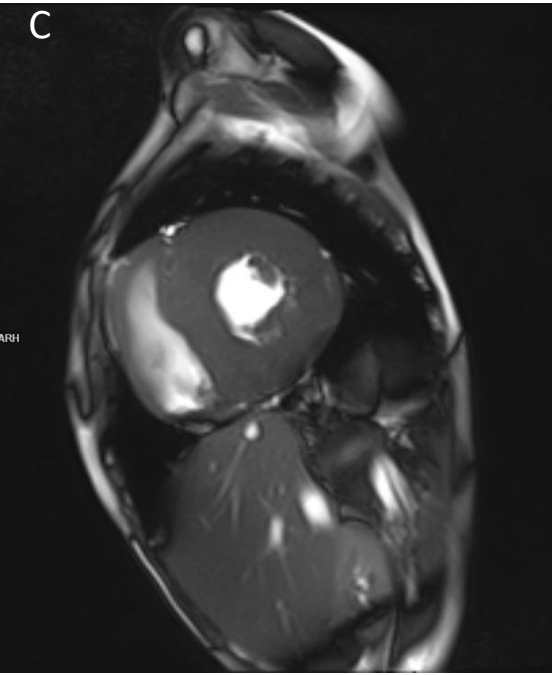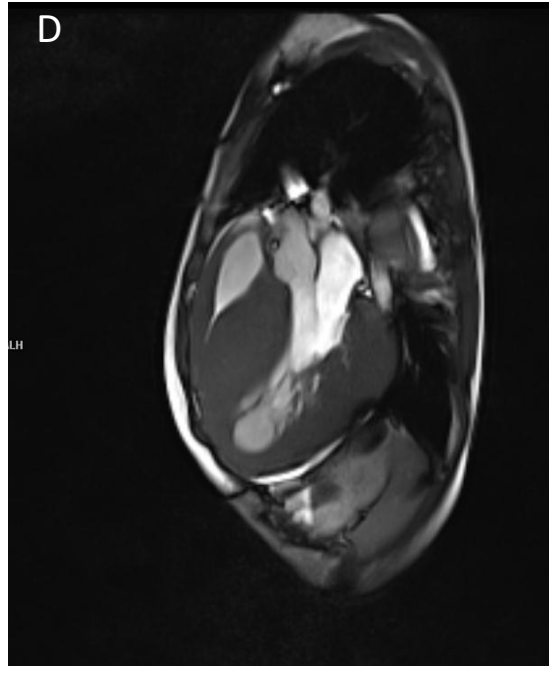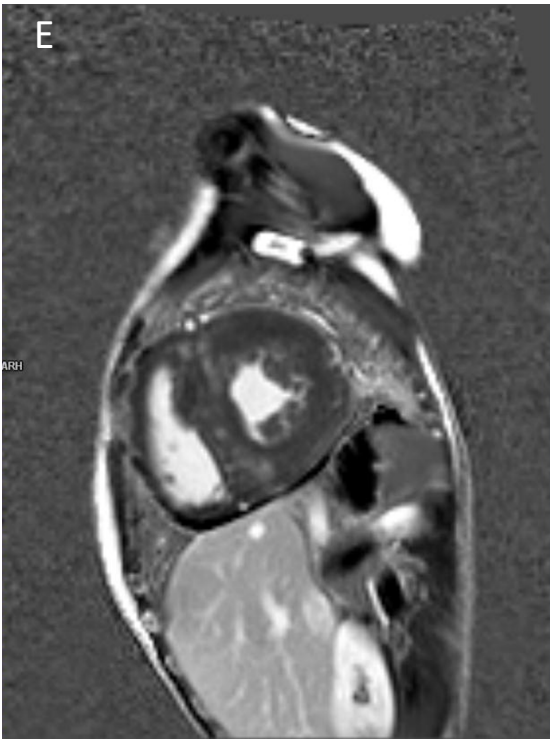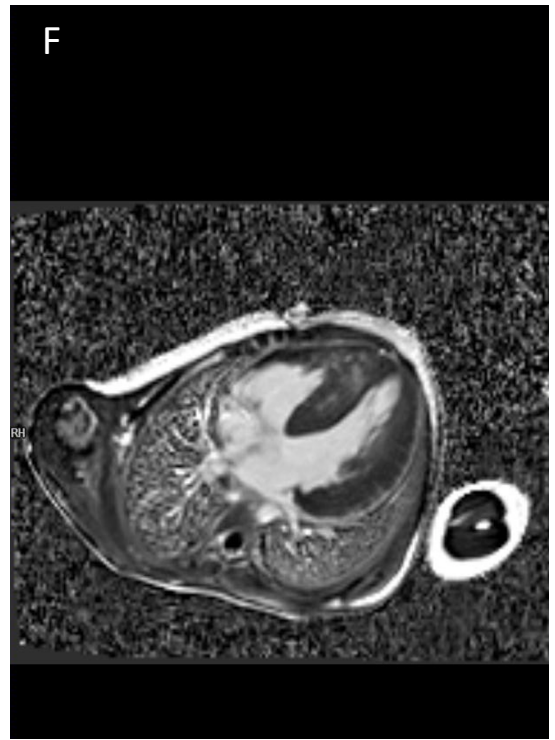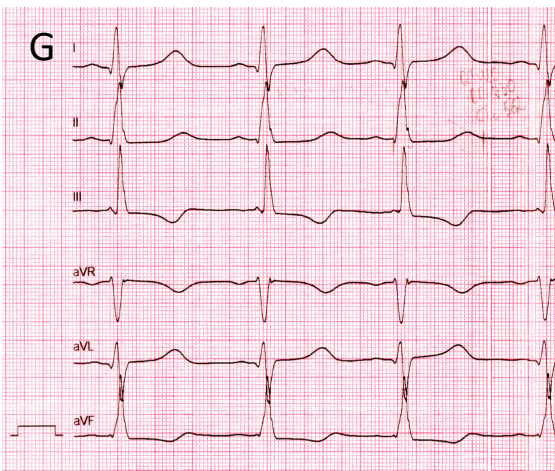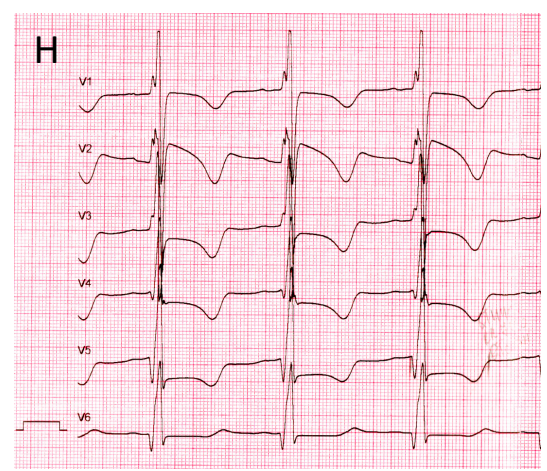

Supplement: Supplementary file 1 [file genes-11-01201-s001.zip › genes-964740- supplementary/ALPK3_genes-proofread/Figure 2.pdf]

Patient 1  
chr15:85383936  
delG

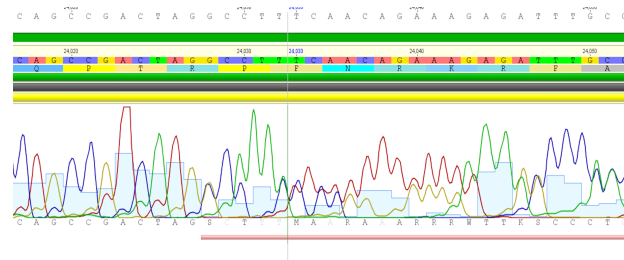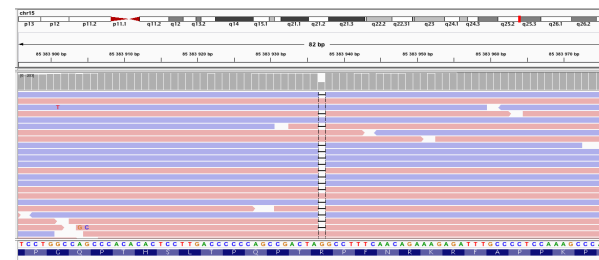

Patient 1  
chr15:85400920  
delG

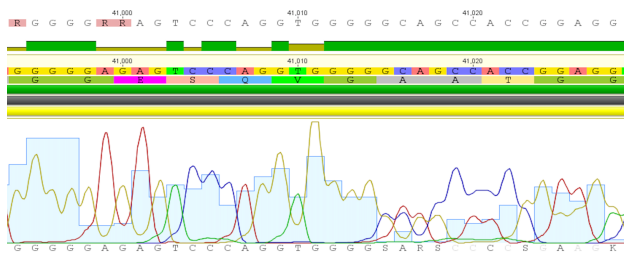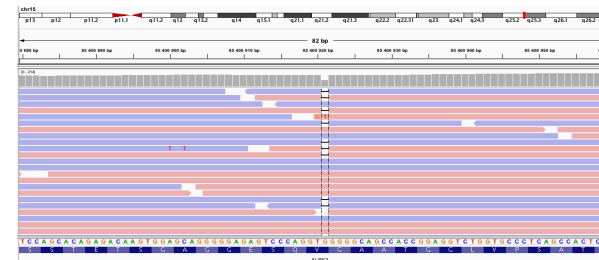

Patient 2  
chr15: 85406027  
G>A (homo)

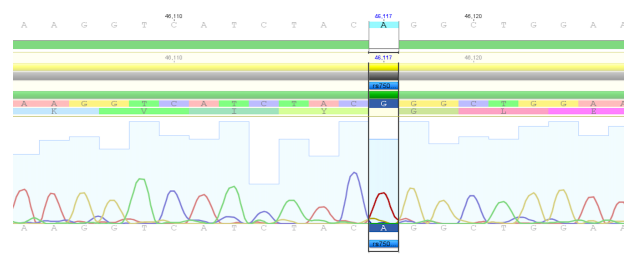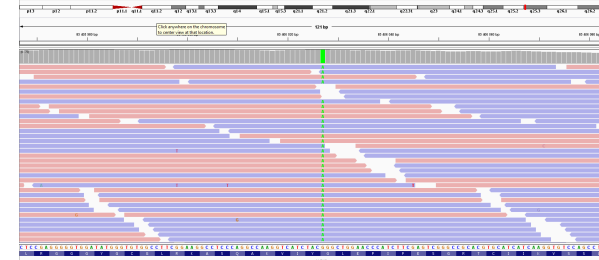

Supplement: Supplementary file 1 [file genes-11-01201-s001.zip › genes-964740- supplementary/ALPK3_genes-proofread/Figure 3.pdf]
